# Ten tips to harnessing generative AI for high-quality MCQS in medical education assessment

**DOI:** 10.1080/10872981.2025.2532682

**Published:** 2025-07-17

**Authors:** Mohi Eldin Magzoub, Imran Zafar, Fadi Munshi, Fouzia Shersad

**Affiliations:** a Department of Medical Education, College of Medicine and Health Sciences, United Arab Emirates University, Al Ain, United Arab Emirates; b National Institute for Health Sciences, United Arab Emirates University, Al Ain, United Arab Emirates; c Department of Medical Education, King Saud bin Abdulaziz University for Health Sciences, Riyadh, Saudi Arabia

**Keywords:** Generative AI, Multiple Choice Questions, Quality, Ten Tips

## Abstract

Generating high quality MCQs is time consuming and expensive. Many strategies are applied to produce high quality items including sharing of item banks, training of item writers and automatic item generation (AIG). Generative AI, when used with precision, has proven to reduce significantly both cost and time without compromising quality. Medical educators encounter numerous obstacles when using AI to generate MCQs of good quality. We searched the fast and recent growing medical education literature for articles related to the use of AI in generating high quality MCQs. Additionally, the development of these tips was guided by our own institutional experience. ** **We created 10 tips for MCQ generation using AI to assist MCQ item writers in both undergraduate and graduate medical education.

## Introduction

Generating a high-quality multiple-choice question (MCQ) in medical education is a time-consuming process. It is estimated that around 24 hours may be needed to generate one high-quality MCQ in medical education, considering the complexities and long process involved in ensuring its validity and reliability. The process of creating multiple-choice questions (MCQs) can be quite expensive, with estimates suggesting that the cost for generating a single item can range from 1,500$ to 2,500$ [1]

The use of pre-structured guidelines has also been proven to enhance the validity and reliability of assessment items [2]. Automatic Item Generation (AIG) has been explored as a viable strategy for creating MCQs that assess a range of cognitive skills, from basic recall to clinical reasoning [3]. While these approaches have yielded positive results, the creation of high-quality MCQs remains a resource-intensive process requiring significant time, cost and commitment. The ultimate goal is to produce assessments that accurately reflect candidates’ abilities. Despite the advancements in improving MCQ quality, time and cost constraints continue to pose challenges for medical educators.

Many strategies have been used by undergraduate and graduate medical education to generate questions of high quality. Prominent among these strategies are sharing of item banks, training of item writers, development of guidelines, policy and regulation and automatic item generation. Sharing of item banks has been proved to be effective in increasing the number as well as the quality of items [4]. Full-fledged Faculty Development Programs (FDPs) have been shown to enhance the skills of medical educators in formulating quality MCQs, resulting in a reduction of flawed items and an increase in questions addressing higher cognitive domain [5]. In some cases, even brief educational interventions have demonstrated short-term improvements in MCQ writing quality, creating a positive impact on the validity of assessments [6].

Another strategy which has been proven to improve validity and reliability is the use of a pre-structured guideline [2]. Automatic Item Generation (AIG) has been explored as a viable strategy for creating MCQs that assess a range of cognitive skills, from basic recall to clinical reasoning (Muse et al., 2023). While these approaches have yielded positive results, the creation of high-quality MCQs remains a resource-intensive process requiring significant time, cost and commitment. The ultimate goal is to produce assessments that accurately reflect candidates’ abilities. Despite the advancements in improving MCQ quality, time and cost constraints continue to pose challenges for medical educators.

Generative AI, when used under precise conditions, has shown to reduce significantly both cost and time without compromising quality. Medical educators encounter numerous obstacles when using AI to generate MCQs of good quality. The integration of generative artificial intelligence (AI), particularly through models like ChatGPT, into the development of assessment items in medical education, offers a promising solution to the challenges of producing high-quality, diverse, and relevant multiple-choice questions (MCQs) efficiently. This suggests that AI-generated items can be effectively used in both low and high-stakes examinations, supporting the validity and reliability of the assessment process [7]. Moreover, the use of generative AI in item development aligns with the current educational objectives, offering the ability to generate personalized feedback and adapt teaching strategies to meet individual student needs [8].

Automated item generation (AIG) and AI-based item generation, while distinct, are closely interlinked in their ability to enhance the efficiency and quality of MCQ development. AIG traditionally relies on predefined algorithms and templates to generate items systematically, ensuring consistency and alignment with educational standards. In contrast, AI-based item generation utilizes advanced machine learning models, like generative AI, to produce items that can adapt more flexibly to diverse educational contexts and content areas. The integration of AI into the item generation processes introduces a new dimension of creativity and adaptability, enabling the generation of items that not only meet technical specifications but also align closely with real-world scenarios and complex cognitive demands [7]. Both methods contribute to reducing the time and cost associated with item generation, yet their interconnection lies in how AI enhances the capabilities of traditional AIG, offering a more dynamic approach to assessment creation.

The potential of AI to transform medical education extends beyond automated item generation, with implications for enhancing the overall teaching and learning experience [9]. However, the successful integration of AI into medical education requires careful consideration of its limitations and the development of consensus-based guidelines to govern its use [10]. Furthermore, it is essential for educators to understand the limitations of AI-generated content, such as potential biases and accuracy issues, and actively shape the development and implementation of these technologies in health professions education.

In conclusion, the utilization of generative AI in the development of assessment items represents a significant advancement in medical education. We have identified specific measures to be taken by item writers which can help them leverage AI to generate high quality MCQs. By applying this knowledge, educators can harness AI tools to enhance the quality, diversity, and relevance of assessment items, making the assessment process more efficient, comprehensive, and aligned with educational objectives [11].

**Figure 1.**
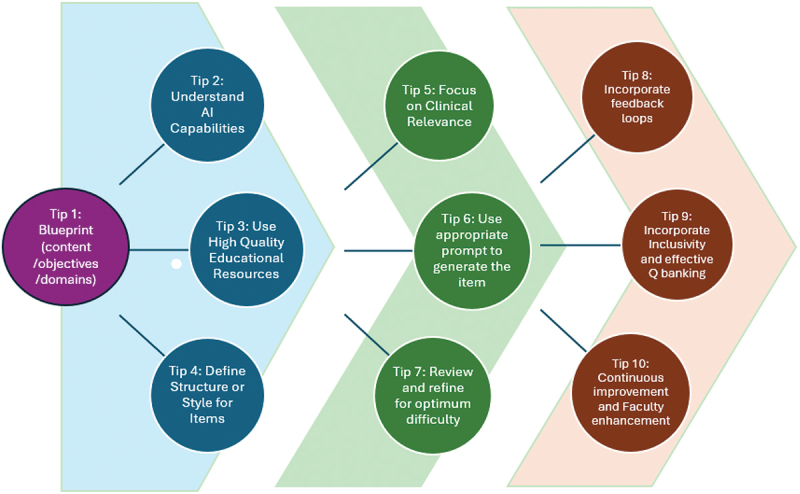
Ten Tips to harnessing generative ai for high quality Mcqs in medical assessments.

From our experience, we have identified the following 10 key tips to be borne in mind when using AI in the item generation process as shown in Figure 1.

As shown in Figure 1, Ten essential tips have been identified for maximizing the efficiency of AI tools in creating assessment items.

### Tip 1: Define learning objectives and blueprint

Item generation through artificial intelligence involves specifying the section of the blueprint and the domain to be tested, so that the AI can generate relevant items. Scholars have reported that content validity – the degree to which a test measures the subject matter it purports to measure- is the most important type of validity for measuring academic achievement [12]. As with any other test-construction process, in AI generation as well, the use of a blueprint is a fundamental and essential first step for clinician educators to ensure high content validity [13]. Assessment blueprints guide the construction of assessment items by identifying key knowledge and skill domains, aligning items with learning objectives, ensuring content validity, and distributing appropriate weightage across topics [14]. Research indicates that a well-crafted blueprint can significantly facilitate the use of generative AI in creating a comprehensive exam with minimal effort [15].

By establishing clear learning goals and a structured plan, educators can guide the AI algorithms to generate test items that align with the desired educational outcomes.

The knowledge to be tested by the item should be communicated to the AI tool using at least two dimensions, which should be included in the prompt for the item generation process. In the example provided in Table 1, the two dimensions are the topic and the domain. This approach ensures that the AI tool can accurately select the desired topic and generate a question aligned with the specified domain.

**Table 1.** An example of a template based on the blueprint from step 1.

PULMONARY DISEASES (13% of exam)	Recognizing Pathophysiology	Making a diagnosis	Ordering Investigations	Interpreting results of tests	Recommending treatment	Assessing risk and prognosis	No of items	Percentage
Obstructive airway disease							5	2.5
Occupational and environmental lung disease							1 to 4	< 2
Restrictive lung disease							1 to 4	< 2
Interstitial lung disease							1 to 4	< 2
Upper respiratory infections							1 to 4	< 2
Lower respiratory infections							1 to 4	< 2
Pulmonary vascular disease							1 to 4	< 2
Pleural disease							1 to 4	< 2
Congenital lung disease							1 to 4	< 2
Sleep medicine							1 to 4	< 2
Evaluation of common pulmonary symptoms: cough, dyspnea, hemoptysis							5	2.5
Solitary pulmonary nodule							1 to 4	< 2

The Table 1 shows an Example of blueprint of the Pulmonary System in Internal Medicine.

Using the example in Table 1, if we choose ‘Lower Respiratory infection’ in the “ordering investigations’ domain, generative AI will create an item assessing this area.

### Tip 2: Understand AI capabilities

Familiarize yourself with the capabilities and limitations of the AI tools you are using. Different AI systems have varying strengths in natural language processing, data analysis, and content generation.

Generative AI and large language models like ChatGPT, have shown promising capabilities in creating effective assessment items in medical education. Even before the advent of AI, the use of automated item generation (AIG) had shown to produce high-quality medical test items that are comparable to manually written items in terms of quality, usability, and validity, offering larger numbers of items with an easy-to-learn process for item development [16]. The integration of generative AI into medical education extends beyond item generation to potentially transforming healthcare education, research, and clinical practice. However, it necessitates the development of consensus-based guidelines to ensure its appropriate use [17]. While generative AI technologies like ChatGPT offer significant potential benefits, including explaining complex topics and creating personalized learning experiences, they also present challenges such as ensuring accuracy, addressing biases, and preventing academic misconduct [2]. These technologies can enhance medical education by providing realistic simulations, personalized feedback, and overcoming language barriers, but they require careful evaluation and management of content quality and ethical concerns [16]. Moreover, AI’s role in medical imaging and the generation of visual explanations highlight its potential to uncover novel scientific discoveries and improve our understanding of health determinants [18].

The rapid advancement of AI in medicine and its implications for assessment integrity and ethical considerations necessitate a careful reconsideration of how assessments are designed and implemented [10,19]. Engaging with AI, as demonstrated in discussions with ChatGPT, reveals its limitations and the need for academic policies to ensure responsible use in education [20]. Ethical concerns about AI’s role in authorship and the integrity of academic work further underscore the importance of developing strategies to integrate AI into medical education responsibly [11].

From our experience, we have identified the following features are required for efficient item generation:

- Ability to upload resources and maximum size of resource which can be uploaded
- Upload time
- Customizability
- Generation time
- Confidentiality and security
- Provision for item regeneration with more prompts
- Provision for review
- Quality of MCQ (overall)
- Accuracy of content and distractors
- Accuracy of resources used
- Number of MCQs generated in one batch
- Technical parameters
- Scalability
- Utilize NLP

We have to l**everage Natural Language Processing (NLP)** capabilities of AI to generate questions that are clear, concise, and grammatically correct. This enhances the readability and comprehension of the assessment items.

### Tip 3: Use high-quality educational resources

Train your AI models on high-quality, peer-reviewed medical literature and evidence based educational resources. This ensures the generated assessment items are accurate and relevant. This requires selection of literature sources which give

To train AI models on high-quality, peer-reviewed medical literature and educational resources, one can follow various approaches outlined in the provided contexts. Firstly, leveraging large language models like GPT-4 can aid in contextualizing training of the AI models to generate realistic exam questions with high accuracy [21]. Additionally, utilizing electronic devices for training AI models by acquiring and labeling data points from datasets can enhance model training [22]. Integrating these methodologies ensures AI models are trained on precise and relevant medical and educational content.

### Tip 4: Define a structure or style for items

Utilizing a standardized structure is crucial to maintain consistency and quality in MCQ generation for medical education. The format and structure should be carefully designed, reviewed and refined to avoid common item writing flaws and ensure visibility of key points. This structure acts as a guideline to ensure that all questions meet educational standards and are consistent in construct thereby increasing validity and reliability.

When generating MCQs through AI, adherence to this standardized structure is essential [7]. Additionally, incorporating principles of generative software development into question creation allows for the generation of single-choice-question-families with varying features and parameters, enhancing the diversity and quality of questions [23].

By ensuring that AI-generated MCQs follow a standard structure, educational institutions can uphold the integrity and effectiveness of their assessment processes; item writers can ensure that AI-generated MCQs are accurate, pedagogically sound, and consistent with educational objectives.

### Tip 5: Focus on clinical relevance

It is imperative to ensure that the assessment items are clinically relevant and reflect authentic and real-world medical scenarios. This helps students to gain skills in clinical reasoning, critical thinking, and problem solving.

AI tools can incorporate real case studies to create realistic and dynamic assessment scenarios. AI can help generate varied and complex cases for comprehensive evaluation. To generate medical multiple-choice questions (MCQs) through AI, a combination of real patient data can be utilized. Synthetic data generation techniques, such as EHR-M-GAN and SyntheX, offer solutions to privacy concerns and data scarcity [24]. These methods can create diverse and realistic patient data, including mixed-type time series EHR data and medical images, which are crucial for training AI models [25] (Sairaj & Balasundaram, 2020) [26]. By leveraging these synthetic datasets along with real patient data, AI algorithms can be trained to develop MCQs based on a wide range of medical scenarios and conditions [27]. This approach ensures the integrity of the data while maintaining classification performance, enabling the creation of high-quality MCQs for medical education and assessment purposes.x

### Tip 6: Using appropriate prompt to generate the item

The development of optimal prompts has been thoroughly researched, and several proposed prompts have been subjected to meta-analysis. It has been observed that the specific requirements, highlighted as key recommendations in this article, have been validated by multiple studies as being critical for generating high-quality items.

Use of customized generative AI specifically for item generation is found to be greatly advantageous. Several AI software solutions now feature customized modules for item generation. These are tailor-made and will help to streamline prompt creation and improve accuracy of the output.

To further enhance the quality of generated items, it is advisable to allocate sufficient processing time for the generative AI system. Users should refrain from modifying or refining prompts before the AI has completed its initial inference. Adequate time has to be allowed for the uploading of resource to be complete before the prompting. Moreover, generating an excessive number of questions or uploading large datasets in rapid succession may lead to biases and errors, such as AI hallucinations, thereby compromising the integrity of the output.

**Different components of an optimum Prompt:**

Several researchers have published prompts which may be used in medical education. Based on the prompt proposed by [28] and [29], we have created an example which may serve as a prototype that can be customized by item writers according to their need.

The prompt used by [29] uses NBME style as the format and makes the prompt easier to manage. The prompt published by Zuckermann is quoted below:

‘Write a multiple-choice question in NBME format with 4 sentence clinical vignette, vital signs, and exam findings. Avoid pseudovignettes. [Item-Specific Test Point as a learning objective or disease state or physical exam finding’ [29]

Zuckermann also illustrates how item writers prevent flaws such as pseudovignettes. Greater accuracy can result with more details in the prompts such as the level of the test-takers and specialization in the case of specialty examinations.

In contrast to the Zuckerman’s prompt, the prompt used by Kiyak is more lengthy and contains the following 9 components

1. Topic in Blueprint -we also added the domain
2. Type of question – e.g., single best answer-type
3. Structure or Framework of the question using a case
4. Details of case a single narrative paragraph with the following points
  - Appropriate age and gender
  - presenting complaints
  - Relevant clinical history
  - Physical examination findings
  - Diagnostic test results (optional)
5. Question stem including a lead in question
6. Answer options should include four distractors and one correct answer.
7. Explanation
  - A detailed explanation for the correct answer
  - Why this is the best option
  - Reasons why each distractor is not the best option
8. The source of the information used for the question and explanations.
9. Difficulty level – Specify the difficulty level as High, Medium, or Low.

By combining the components of the two prompts, the authors have created a prompt to create an example item. Find below a follow-up of an actual prompt and the item generated by ChatGPT by the researchers. This has been generated for the purpose of illustrating the process by which the above points can be used.

**The prompt used by the authors for this example:**

“Please generate a single best answer multiple choice question around a patient with community-acquired pneumonia, targeting the domain of investigations to be ordered for diagnosis.

This should be an NBME-style multiple-choice question (MCQ) designed to test a final-year resident using a complex case scenario. The question should be a single best answer type with one correct option and three plausible distractors (not True/False).

The question stem should include the following patient details:

Appropriate age and gender
Relevant clinical features
Clinical history
Physical examination findings
Diagnostic test results

Additionally, please provide:

A detailed explanation for the correct answer
Reasons why each distractor is incorrect
The source of the information used for the question and explanations.

The difficulty level should be (High, Medium, or Low).”

**Example of the Question generated by Generative Artificial Intelligence**

“A 65-year-old male with a 40-pack-year smoking history presents to the emergency department with a 3-day history of fever, productive cough with yellowish sputum, and pleuritic chest pain. He also reports feeling increasingly short of breath. He has no history of recent hospitalizations. Physical examination reveals a temperature of 38.5°C (101.3°F), heart rate of 105 beats per minute, respiratory rate of 22 breaths per minute, and blood pressure of 130/85 mmHg. Auscultation of the lungs reveals decreased breath sounds and crackles in the right lower lung field. A chest X-ray shows consolidation in the right lower lobe.

Which of the following is the most appropriate initial diagnostic investigation to confirm the diagnosis of community-acquired pneumonia in this patient?

- Blood cultures
- Sputum Gram stain and culture
- Chest computed tomography (CT) scan
- Arterial blood gas (ABG) analysis

Correct Answer: B. Sputum Gram stain and culture

**Explanation**:

- Option B (Correct): Sputum Gram stain and culture is the most appropriate initial diagnostic investigation in this case. It helps identify the causative organism and guide antibiotic therapy, which is crucial in managing community-acquired pneumonia (CAP).
- Option A (Incorrect): Blood cultures are useful in detecting bacteremia but are not the first-line diagnostic test for pneumonia. They are more appropriate in severe cases or when the patient is hospitalized.
- Option C (Incorrect): A chest CT scan provides detailed imaging but is not necessary for the initial diagnosis of CAP, which can be diagnosed with a clinical examination and chest X-ray. CT is reserved for complicated cases or when there is suspicion of an underlying condition not visible on a standard X-ray.
- Option D (Incorrect): Arterial blood gas (ABG) analysis is useful for assessing respiratory status, particularly in severe cases with hypoxemia, but it does not confirm the diagnosis of pneumonia.

Source: This question and explanations are based on the clinical guidelines for the management of community-acquired pneumonia as outlined by the Infectious Diseases Society of America (IDSA) and the American Thoracic Society (ATS).”

This item is generated from the part of the blueprint as required by the prompt. It is sensibly constructed and it complies with the required structure that was specified in the prompt. This may be improved by more iterations, refining the prompt to achieve the desired level of clarity and difficulty.

### Tip 7: Review and refine through human oversight

Human oversight is necessary to guarantee accuracy, relevance, and congruence with learning goals. The reviewers have to be trained and the AI-generated assessment items should be reviewed and improved to ensure their quality and relevance [30]. Regular reviews should be conducted to enhance the items’ psychometric characteristics, reliability, and validity, potentially through pilot-testing to gather statistical information for further refinement [31]. Quality must be evaluated firstly by subjecting items created by AI to be rated by a two- to four-member expert medical panel. The panel should include content as well as process experts in item writing and review.

The first level of review is the technical review. The parameters of review should include all components such as the stem, the lead-in question, and options and their qualities (homogeneity, duplication, redundancy, ambiguity and logical progression). This also includes checking for technical flaws such as wrong format and presence of unintended clues to the correct answer. This review may use a checklist as shown in Table 2 or similar quality check instrument.

**Table 2.** Checklist for reviewing the items generated by AI.

		Yes	No
1.	Is the stem stated as a direct question? *(Can you guess the options/correct answer of the MCQ?)*	✓	
2.	Is there any unnecessary information in the stem?		✓
3.	Are all options homogenous? *(e.g., muscles, diagnosis, lab tests)*	✓	
4.	Are there any unnecessary repeats in the options?		✓
5.	Are options in logical order? *(e.g., chronological, most to least, alphabetical)* Also make sure there is no grouping errors in the options.	✓	
6.	Are there any opposite options?		✓
7.	Are there any ineffective options?		✓
8.	Is there any relatively long option? *(correct or incorrect)*		✓
9.	Are there any unintended clues in the stem/options?		✓
10.	Are stem and option texts clear and unambiguous? *(Does NOT containing vague or absolute words e.g., usually, frequently, never, always)* Also check grammar errors/typos in the stem and options.	✓	
11.	Is ALL of ABOVE/NONE of ABOVE used as an option?		✓
12.	Is the stem phrased NEGATIVELY?		✓

* Partially adapted from NBME® ITEM-WRITING GUIDE Constructing Written Test Questions for the Health Sciences, (2021) National Board of Medical Examiners https://info.nbme.org/item-writing-guide.html.

The next level of review and refining process is targeted at **ensuring optimum level of question difficulty**. To generate questions with varying difficulty levels for students, cognitive models like Bloom’s Taxonomy and Item Response Theory (IRT) can be utilized. Bloom’s Taxonomy aids in categorizing questions based on cognitive levels [32] while IRT considers learners’ proficiency to assign difficulty levels to questions [33]. Additionally, a six-component model for problem-solving assessment can help identify key concepts and skills crucial for challenging students appropriately [31]. By incorporating these models, educators can adjust the complexity of problems, or the information required to solve them, catering to different proficiency levels and enhancing the learning experience for students. Additional complexity may be achieved by adding a comorbidity such as renal failure or withholding data. In some cases, modifying laboratory values or introducing conflicting psychosocial elements may raise the level of the item to a graduate level question. By modifying the distractors to be more plausible, the difficulty level can be enhanced. This approach ensures that questions are tailored to students’ abilities and promotes critical thinking and skill development in various educational settings.

### Tip 8: Incorporate feedback loops

AI systems in medical education assessment can benefit significantly from student feedback. By utilizing AI models like Chat Generative Pre-Trained Transformer [11], AI can provide near-instantaneous access to comprehensive information and individualized feedback to students, enhancing engagement and learning outcomes. Additionally, AI tutoring systems can offer metric-based assessment and formative feedback on quantifiable criteria and actionable goals, proving more effective than expert instruction [34]. Moreover, Natural Language Processing (NLP) algorithms can automatically categorize narrative feedback into specific competencies, guiding trainee progress and self-assessment [35]. AI models can also be developed to analyze student performance in specific skills through video-based assessments, providing automated ratings and specific feedback to enhance skill acquisition [36]. Overall, AI systems leverage student feedback to improve assessment accuracy, guide learning, and enhance educational outcomes in medical education.

### Tip 9: Ensure inclusivity and effective question banking

Create assessment items that are inclusive and free from bias. AI models should be trained on diverse datasets to avoid cultural, gender, or racial biases.

AI can aid in generating diverse medical multiple-choice questions (MCQs) by leveraging question generation models that incorporate visual and language information in different latent spaces to ensure diversity [19]. Additionally, combining variational autoencoders with long short-term memory networks can facilitate the creation of a large set of varied questions from a single input image, showcasing the potential for creativity in question generation tasks [16]. The questions generated through the aforementioned steps should be piloted in actual exams as additional items. These items should be reviewed for difficulty and psychometric properties. Questions that meet quality standards should be archived in a question bank for future use.

### Continuous improvement and faculty enhancement

Keep updating the AI models with new medical knowledge and educational trends. Medical education is an evolving field, and AI systems should stay current to provide relevant assessment items.

To continuously improve and enhance faculty while generating medical MCQs through AI, collaboration among educators, researchers, and practitioners is crucial. Additionally, adopting Appreciative Inquiry (AI) principles in medical education can further enhance faculty development by focusing on strengths and positive aspects, fostering a generative process for envisioning valued future situations [37]. By integrating these strategies, educators can ensure ongoing research, interdisciplinary collaboration, and the ethical use of AI-generated content, contributing to enhanced learning experiences and faculty development in medical education.

Faculty can enhance medical MCQ generation through AI by leveraging generative language models (GLMs) for realistic simulations, personalized feedback, and evaluation methods [37]. Additionally, continuous quality improvement (CQI) can play a vital role in refining the MCQ generation process by enhancing reflective functions and response times to important issues [38]. However, challenges such as ensuring content quality, addressing biases, and managing ethical concerns need to be mitigated to optimize AI’s potential in medical education. Implementing AI in MCQ generation requires collaboration among educators, researchers, and practitioners to develop guidelines and policies for responsible use [39].

## Conclusion

AI-powered educational assessment tools offer numerous benefits through following the above logical and sequential tips to generate and improve the accuracy, efficiency and cost effectiveness of MCQs production. Additionally, generation of personalized feedback for students, and enabling teachers to adapt their teaching strategies to meet the unique needs of each student. However, while AI presents promising opportunities for the future of medical education assessment, particularly production of high-quality MCQs, it is also accompanied by challenges and limitations that must be addressed. These include the need for further research to refine AI’s role in assessment to establish clear foundations for its integration into medical education assessments and generation of MCQs. Moreover, the ethical use and potential unintended consequences of AI in medical education necessitate careful consideration and the development of academic policies to ensure responsible utilization. In conclusion, AI, particularly models like Chat-GPT and GPT-4, offers promising opportunities to enhance production of high quality MCQs. However, the successful integration of these technologies requires a balanced approach that considers both their potential benefits and limitations, supported by ongoing research and ethical considerations.
